# Examining the Role of Hypothalamus-Derived Neuromedin-U (NMU) in Bone Remodeling of Rats

**DOI:** 10.3390/life13040918

**Published:** 2023-03-31

**Authors:** Gabriella Born-Evers, Ashley L. Orr, Elizabeth Q. Hulsey, Maria E. Squire, Julia M. Hum, Lilian Plotkin, Catherine Sampson, Jonathan Hommel, Jonathan W. Lowery

**Affiliations:** 1 Division of Biomedical Science, College of Osteopathic Medicine, Marian University, 3200 Cold Spring Rd, Indianapolis, IN 46222, USA; 2 Bone & Muscle Research Group, Marian University, Indianapolis, IN 46222, USA; 3 Department of Biology, The University of Scranton, Scranton, PA 18503, USA; 4 Department of Anatomy, Cell Biology & Physiology, Indiana University School of Medicine, Indianapolis, IN 46222, USA; 5 Indiana Center for Musculoskeletal Health, Indiana University School of Medicine, Indianapolis, IN 46222, USA; 6 Department of Pharmacology and Toxicology, University of Texas Medical Branch, Galveston, TX 77555, USA; 7 Division of Academic Affairs, Marian University, Indianapolis, IN 46222, USA

**Keywords:** osteoblast, bone, Neuromedin U, NMU

## Abstract

Global loss of the neuropeptide Neuromedin-U (NMU) is associated with increased bone formation and high bone mass in male and female mice by twelve weeks of age, suggesting that NMU suppresses osteoblast differentiation and/or activity in vivo. NMU is highly expressed in numerous anatomical locations including the skeleton and the hypothalamus. This raises the possibility that NMU exerts indirect effects on bone remodeling from an extra-skeletal location such as the brain. Thus, in the present study we used microinjection to deliver viruses carrying short-hairpin RNA designed to knockdown *Nmu* expression in the hypothalamus of 8-week-old male rats and evaluated the effects on bone mass in the peripheral skeleton. Quantitative RT-PCR confirmed approximately 92% knockdown of *Nmu* in the hypothalamus. However, after six weeks, micro computed tomography on tibiae from *Nmu*-knockdown rats demonstrated no significant change in trabecular or cortical bone mass as compared to controls. These findings are corroborated by histomorphometric analyses which indicate no differences in osteoblast or osteoclast parameters between controls and *Nmu*-knockdown samples. Collectively, these data suggest that hypothalamus-derived NMU does not regulate bone remodeling in the postnatal skeleton. Future studies are necessary to delineate the direct versus indirect effects of NMU on bone remodeling.

## 1. Introduction

Osteoblasts are cells of the skeleton that produce bone matrix while osteoclasts are cells that resorb bone matrix [1]. The balance of activity between these cell types determines bone mass. Osteoporosis is a disease of low bone mass that increases an individual’s risk for fractures and resulting disability and/or mortality. Hospitalizations due to osteoporotic fractures in the US are greater than the number of hospitalizations for myocardial infarctions, strokes, and breast cancer [2]. Osteoporosis is a chronic condition and treatment strategies generally focus on building bone mass. That said, there are few pharmacologic treatment options for long-term management due to important contraindications and side effects. Thus, identifying novel therapeutic opportunities aimed at increasing bone mass is an important goal. 

Neuromedin-U (NMU) is an evolutionarily conserved peptide that is expressed in numerous anatomical locations. In addition to participating in such varied physiologic actions as blood pressure regulation, stress responses and feeding behavior [3,4,5], strong genetic evidence from two independent studies implicates a functional role for NMU in the regulation of bone remodeling in vivo. Both Sato et al. and Hsiao et al. report that global loss of NMU expression in mice is associated with high bone mass due to increased bone formation rate with no alteration in osteoclast parameters [6,7]. Additionally, exogenous administration of NMU peptide to osteoprogenitor cells in vitro is associated with reduced expression of the osteoblastic markers *Sp7*/*Osterix* and *Bglap*/*Osteocalcin* [7]. Taken together, these findings suggest that NMU is an endogenous repressor of osteoblast differentiation and/or activity and presents an attractive opportunity for inhibiting NMU function as a means of increasing bone mass. However, it is unclear where NMU signals to carry out this action since, although NMU is expressed in the skeletal microenvironment and acts directly on osteoblasts [7,8,9], it also has a broad expression pattern including strong expression in the hypothalamus. Furthermore, continuous intracerebroventicular (ICV) delivery of NMU into the third ventricle for four weeks reduces bone mass of the tibia [6], indicating that NMU is capable of regulating bone remodeling in the appendicular skeleton through actions in the central nervous system.

Thus, in the present study, we knocked down endogenous expression of NMU in the hypothalamus of adult rats using short-hairpin RNA and, after five weeks, evaluated for effects on osteoblast and/or osteoclast number and changes in bone mass of the appendicular skeleton. Despite efficient reduction in *Nmu* mRNA levels (>90%), we found no alterations in bone metabolism by quantitative histomorphometry and computed tomography analyses. This leads us to conclude that hypothalamus-derived NMU does not regulate bone remodeling in the postnatal skeleton. Future studies are necessary to delineate the direct versus indirect effects of NMU on bone remodeling.

## 2. Materials and Methods

### 2.1. Rats

Long–Evans blue spruce outbred male rats (HsdBlu:LE, Envigo, Houston, TX, USA) weighing 225–250 g and approximately 8 weeks of age were single housed and allowed to habituate for 7 days prior to stereotaxic surgery. Animals were divided into two groups of 8 and received AAV-shNMU or AAV-shCTRL as described below. One week post-surgery, rats were switched from a standard brown chow (Teklad Mouse/Rat Diet 7912, Harlan) to a high fat diet as previously described [10]. The high fat diet was used since this is associated with increased hypothalamic NMU expression, thus providing a preferable scenario in which to examine the NMU regulation of bone remodeling. Animals were maintained on their respective diets for 35 days, with body weight and food intake measured daily. All procedures were performed in accordance with the Institutional Animal Care and Use Committee of the University of Texas Medical Branch (protocol #1701005A).

### 2.2. Hairpin Design

Hairpin RNA was designed to target *Nmu* rat mRNA as previously described [10]. Briefly, we identified seven different 24-nucleotide sequences within Ensembl:ENSRNOG00000002164. The oligonucleotide sequences (Sigma-Aldrich, St. Louis, MO, USA) were synthesized as follows: shNMU-1 top 5′-TTTGAACCTCTTAGCATTATCTTTCTCCTTCCTGTCAGAGAAAGATAATGCTAAGAGGTT-3′ and shNMU-1 bottom 5′-CTAGAAAAATAACCTCTTAGCATTCTCTTTCTCTGACAGGAAGGAGAAAGATAATGCTAA-3′. The pAAV-shRNA and control hairpin (shCTRL) plasmid was described previously [10]. RNAi viruses were produced using a triple-transfection, helper-free method and purified as previously described [11].

### 2.3. Cell Culture

Human Embryonic Kidney 293 (HEK293) cells were selected for in vitro screening experiments. All cells were cultured in a humidified incubator at 37 °C in 5% CO_2_, up to passage 25, in complete media, containing Dulbecco’s modified Eagle’s medium (DMEM) (cat no. 31053028, Gibco, Carlsbad, CA, USA), supplemented with 10% Fetal Bovine Serum (FBS) (cat no. 16000044, Gibco, Carlsbad, CA, USA), 100 U/mL penicillin and 100 µg/mL streptomycin (cat no. BP295950, Fisher BioReagents, Pittsburg, PA, USA).

### 2.4. In Vitro Hairpin Screening

Knockdown efficacy was tested in vitro using HEK-293 cells with six pAAV-shNMU hairpin vectors and one pAAV-shCTRL vector. To create an *Nmu* expression vector, the rat *Nmu* gene sequence (NCBI NM_022239.2) was synthesized (GenScript, Piscataway, NJ, USA), then cloned into the pAAV-MCS expression plasmid (Stratagene, San Diego, CA, USA). For hairpin screening, HEK-293 cells were transfected and real-time PCR performed as previously described [10].

### 2.5. Microinjection Surgery

Microinjection surgery was performed as detailed in a prior report [10]. Briefly, rats approximately 60 days in age were maintained under anesthesia with an isoflurane vaporizer system (VetEquip, Livermore, CA, USA), shaved, and secured on a stereotaxic apparatus (Kopf, Miami, FL, USA). Then, a small scalp incision was made down the midline to expose the skull and bilateral holes were drilled through the skull above the injection sites. Coordinates for the LH injection site were previously reported [10]. Microinjection of 1μL AAV into the LH on each side was performed at a rate of 0.2 μL every minute for 5 min. Post-injection, the needles remained in place for 5 min and then were removed. Post-surgical care and procedures were as previously described [10]. Rats were allowed to recover for at least 2 weeks post-surgery before starting experiments.

### 2.6. In Vivo Hairpin Screening

After surgery recovery, rats were anesthetized (16 days post viral injection) using the before mentioned isoflurane vaporizer system. Brains were collected and fresh frozen in dry ice for 30 min, before being placed in 50 mL conical tubes and stored at −80 °C for storage. Coronal sections (10 µm) containing the LH (−1.32 through −1.93 from Bregma) were taken from rat brains using a cryostat (Leica CM 1850 at −20 °C) according to the atlas of Paxinos and Watson (Paxinos and Watson 2006). Slices were immediately mounted on uncharged slides (Fisherbrand™ Premium Frosted Microscope Slides, Fisher Scientific, Pittsburgh, PA, USA).

### 2.7. Laser Capture Microdissection

Laser capture microdissection (LCM) was used to select and remove AAV-shNMU infected LH cells from the slices collected on the slides previously mentioned in the in vivo hairpin screening section. Infected cells were identified by detection of eGFP in the LH. LCM was also used to collect infected cells in the AAV-shCTRL group. RNA from the cells was isolated using the appliedbiosystems^TM^ PicoPure^TM^ RNA Isolation Kit (cat no. KIT0204, Applied Biosystems, Waltham, MA, USA). RNA was synthesized into cDNA using the previously described method and materials. Quantitative reverse transcription PCR (RT-qPCR) analysis looking at *Nmu* and *Gapdh* levels was performed as described above.

### 2.8. Immunohistochemistry

Rats were anesthetized with a combination of ketamine and xylazine, euthanized by cardiac puncture, and transcardially perfused for 5 min with 1XPBS (75 mL), followed by 15 min of 4% paraformaldehyde in 1XPBS (225 mL). Brains were then frozen with dry ice; using a microtome (Leica SM 2010R), 40 μm coronal slices containing the LH were collected and stored in vials of 0.01% sodium azide in 1XPBS at 4 °C until use. Immunohistochemistry staining for GFP was conducted as previously described [10,12]. Slices were mounted onto Superfrost Plus microscope slides and allowed to dry overnight. Slides were rehydrated and cover-slipped according to previously published protocols [10,12]. Targeting for each rat was assessed by examining immune-enhanced GFP expression under a confocal microscope. All images for targeting were captured using Leica True Confocal Scanner SPE and Leica Application Suite Advanced software (Leica Microsystems, Germany) [12].

### 2.9. Micro Computed Tomography (µCT)

Tibiae and femora from scramble control and *Nmu*-KD rats were scanned using a high-resolution (10 μm/voxel) μCT 80 scanner (Scanco, Wayne, PA, USA) as previously described and according to established guidelines [8,13].

### 2.10. Histomorphometry

Histological preparation and histomorphometic analyses were performed on distal femora as previously described [8].

### 2.11. Statistical Analyses

Data were plotted in GraphPad Prism 8 (San Diego, CA, USA). Normal distribution was tested using Shapiro–Wilk test and statistical significance between groups determined using unpaired *t* test, Mann–Whitney test, or one-way ANOVA with Bonferroni post hoc correction as detailed in the legend; *p* < 0.05 was considered significant.

## 3. Results

### 3.1. Validation of NMU Knockdown

**Figure 1:**
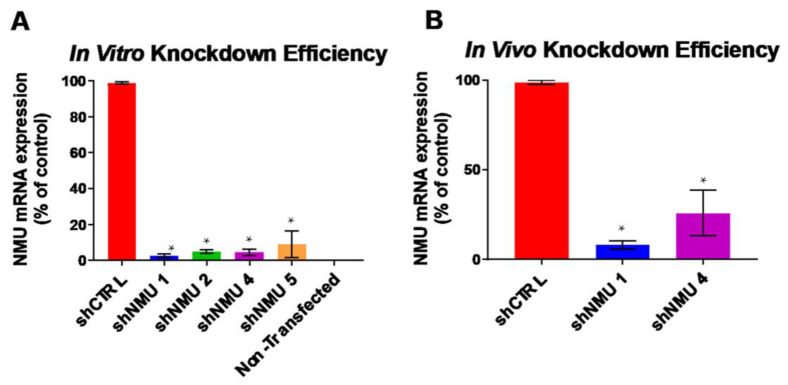
Targeting and knockdown efficiency of *Nmu*. (**A**) 90–95% knockdown of in vitro *Nmu* in HEK293 cells is accomplished using each of the shNMU hairpins tested. shNMU 1 and shNMU 4 had the highest in vitro knockdown percentages at 97.4% and 95.4%, respectively. (**B**) In vivo knockdown of endogenous *Nmu* in the LH is achieved using AAV-shNMU, with AAV-shNMU 1 knocking down ~92% of *Nmu* in the LH. Means ± SEM. for independent experiments (n = 3) rep-resented in (**A**,**B**). * indicates *p* < 0.05 by one-way ANOVA with Bonferroni correction against shCTRL.

We designed 10 shRNA *Nmu* hairpins to investigate the effect of lateral hypothalamic (Figure S1) NMU knockdown on food intake, body weight, and adiposity. Seven of the hairpins were screened for their ability to knockdown *Nmu* in vitro. HEK-293 cells were cultured to ~70% confluency then co-transfected with an *Nmu* expression plasmid, to elucidate *Nmu* expression in the cells, and a shNMU expression plasmid. In vitro experiments were conducted in 6-well tissue culture dishes in replicates of three. Cells were collected from each well and RT-qPCR was used to quantify the amount of NMU in the cells. Three of the seven hairpins (shNMU 1, shNMU 2, and shNMU 4) had a knockdown efficiency greater than 95%, compared to the non-silencing shCTRL (Figure 1A). shNMU 1 and shNMU4 had the highest knockdown percentages, 97.4% and 95.4%, respectively, and were packaged into an adeno-associated virus 2 (AAV2) for in vivo studies.

In vivo knockdown of *Nmu* in the LH was quantified using LCM. Nine animals (n = 3 per group) were transfected with one of the three viruses, AAV-shNMU 1, AAV-shNMU 4, or AAV-shCTRL. The AAV viral vector contains a GFP tag, making identification of infected neurons visible in fluorescent microscopes. All viruses produced detectable amounts of GFP in the LH (Figure 1B), signifying infection. The GFP-positive individual neurons were then collected onto the LCM caps, from which the RNA was isolated. Quantification of *Nmu* knockdown was once again determined using RT-qPCR. shNMU 1 was shown to knockdown ~92% of endogenous *Nmu*, while shNMU 4 knocked down ~75% of endogenous *Nmu* relative to control (Figure 1B). Based on these data, the studies utilizing LH *Nmu* knockdown (*Nmu*-KD) in this paper were conducted using the shNMU 1 virus.

### 3.2. Bone Volume Is Unchanged in the Absence of Hypothalamic NMU

**Figure 2:**
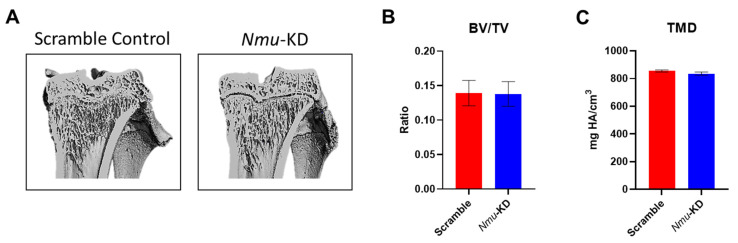
Bone mass phenotype of scramble control and *Nmu*-KD rats. (**A**) Representative µCT images from proximal regions of tibiae from scramble control (**left**) and *Nmu*-KD rats (**right**). (**B**–**C**) Bone volume fraction (**B**), expressed as ratio of bone volume (BV) to tissue volume (TV), and tissue mineral density (TMD, (**C**)) in the trabecular regions of tibiae from scramble control (scramble) and *Nmu*-KD rats. n = 8 for scramble control and *Nmu*-KD rats (n = 6). Data are mean ± SEM and confirmed to be normally distributed using Shapiro–Wilk test. Statistical analysis by unpaired *t* test yielded *p* > 0.05.

Knockdown of *Nmu* had no impact on total weigsht gain or average daily weight gain (data not shown). µCT analyses on proximal metaphyseal region of the tibiae of scramble control and *Nmu*-KD rats revealed that the trabecular bone volume fraction (BV/TV) and tissue mineral density (TMD) in *Nmu*-KD rats are comparable to controls (Figure 2). Consistent with this, trabecular number (Tb.N), trabecular thickness (Tb.Th), trabecular separation (Tb.Sp), and trabecular connectivity density (Conn.D) did not differ between controls and *Nmu*-KD samples (Table 1).

We also performed µCT on the mid-diaphyseal region of tibiae from control and *Nmu*-KD rats. Consistent with results from the trabecular region, cortical bone volume fraction (BV/TV) and cortical thickness (Ct.Th) were comparable between scramble control and *Nmu*-KD samples (Table 2).

### 3.3. Histological Analyses of NMU-KD Bones

Quantitative histological analyses on the distal metaphyseal region of femora from scramble control and *Nmu*-KD rats revealed that osteoblast number, osteoblast surface, and osteoid surface were unchanged by the loss of hypothalamus-derived NMU (Table 3). Similarly, osteoclast number, osteoclast surface, and eroded surface were comparable between scramble control and *Nmu*-KD rats (Table 3). Taken together, these data are consistent with the µCT results.

## 4. Discussion

In the present study, we knocked down endogenous expression of NMU in the hypothalamus of adult male rats using short-hairpin RNA and, after six weeks, evaluated for effects on osteoblast and/or osteoclast activity and changes in bone mass of the appendicular skeleton. Despite efficient reduction in *Nmu* mRNA levels (>90%), we found no alterations in bone metabolism by quantitative histomorphometry and computed tomography analyses. The most direct interpretation of these findings is that hypothalamus-derived NMU does not regulate bone remodeling in the postnatal skeleton.

That said, our study has several important limitations such as the timing and length of knockdown. We chose to knockdown *Nmu* expression in young adult rats to avoid the potential for developmental defects associated with hypothalamic expression of NMU. Similarly, we chose to evaluate bone parameters after six weeks of knockdown since Sato et al. observed that just four weeks of ICV delivery of NMU is sufficient to reduce bone mass in male and female mice. However, it is possible that absence of hypothalamic NMU at an earlier (or later) time point would reveal changes in bone formation. Additionally, we are presently unable to eliminate the possibility that another ligand is capable of compensating for the absence of hypothalamus-derived NMU. For instance, like NMU, Neuromedin-S (NMS) is expressed in the hypothalamus and activates the receptors NMUR1 and NMUR2. However, we do not favor compensation by NMS as a likely explanation since global NMU knockout mice have high bone mass, indicating that NMS does not adequately compensate for NMU in that experimental condition. Thus, the biological source of NMU in the regulation of bone remodeling remains an open question. Work by us and others indicates that NMU is expressed in the postnatal bone microenvironment, raising the possibility that NMU acts as a local regulator of osteoblast function. Consistent with this idea, studies of isolated bone cells reveal that exogenous NMU regulates osteoblast differentiation (with suppression of master osteoblastic differentiation factor *Sp7*/*Osterix*), proliferation, and activity with signaling effects through multiple signaling pathways including mTOR [7,8,9]. However, since a conditional knock-out allele for *Nmu* is not presently available, we are unable to test this hypothesis directly at the present time. Thus, future studies are necessary to delineate the direct versus indirect effects of NMU on bone remodeling.

## Figures and Tables

**Table 1:** µCT analyses from trabecular regions of tibiae from scramble control (n = 8) and *Nmu*-KD rats (n = 6). Data are mean ± SEM. *p* values determined by Mann–Whitney test. Tb.N, trabecular number. Tb.Th, trabecular thickness. Tb.Sp, trabecular separation. Conn.D, connectivity density. TMD, tissue mineral density. #, number.

	Parameter	Scramble Control	*Nmu*-KD	*p* Value
Tibiae	BV/TV (ratio)	0.139 ± 0.018	0.138 ± 0.018	0.96
	Tb.N (#/mm)	2.924 ± 0.200	3.166 ± 0.434	0.99
	Tb.Th (mm)	0.075 ± 0.002	0.074 ± 0.004	0.23
	Tb.Sp (mm)	0.341 ± 0.029	0.317 ± 0.033	0.95
	Conn.D (#/mm^3^)	48.7 ± 7.2	66.9 ± 24.5	0.95
	TMD (mg HA/cm^3^)	854.518 ± 5.577	835.526 ± 11.546	0.13

**Table 2:** µCT analyses from midshaft region of tibiae from scramble control (n = 8) and *Nmu*-KD rats (n = 6). Data are mean ± SEM and confirmed to be normally distributed by Shapiro–Wilk test. *p* values determined by unpaired *t* test. TV, tissue volume. BV/TV, bone volume fraction. Ct.Th, cortical thickness. MaV, marrow cavity volume.

	Parameter	Scramble	*Nmu*-KD	*p* Value
Tibiae	TV (mm^3^)	5.49 ± 0.22	5.49 ± 0.17	0.98
	BV/TV (ratio)	0.975 ± 0.001	0.972 ± 0.002	0.13
	Ct.Th (mm)	0.639 ± 0.012	0.583 ± 0.028	0.10
	MaV (mm^3^)	2.01 ± 0.14	2.44 ± 0.35	0.28

**Table 3:** Histomorphometric analyses from trabecular region of femora from scramble control (n = 5) and *Nmu*-KD rats (n = 5). Data are mean ± SEM and confirmed to be normally distributed by Shapiro–Wilk test. *p* values determined by unpaired *t* test. N.Ob, osteoblast number. B.Pm, bone perimeter. Ob.S, osteoblast surface. BS, bone surface. OS, osteoid surface. N.Oc, osteoclast number, Oc.S osteoclast surface. ES, eroded surface.

	Parameter	Scramble	*Nmu*-KD	*p* Value
Osteoblast Analyses	Osteoblast Number (N.Ob/B.Pm)	49.39 ± 7.42	47.62 ± 2.30	0.83
	Osteoblast Surface (Ob.S/BS)	41.38 ± 5.14	41.07 ± 1.81	0.96
	Osteoid Surface (OS/BS)	16.22 ± 3.47	15.66 ± 1.82	0.89
Osteoclast Analyses	Osteoclast Number (N.Oc/B.Pm)	2.61 ± 1.07	2.57 ± 0.63	0.95
	Osteoclast Surface (Oc.S/BS)	9.97 ± 0.60	10.38 ± 2.67	0.88
	Eroded Surface (ES/BS)	11.06 ± 0.78	10.94 ± 2.76	0.97

## Data Availability

Datasets used and/or analysed are available from the corresponding author on reasonable request.

## Supplementary Materials

The following information is available online at: https://www.mdpi.com/article/10.3390/life13040918/s1, Figure S1: Targeting and knockdown efficiency of *Nmu* in the LH. (**A**) The lateral hypothalamus has previously been identified as a source of *Nmu* for the PVN and is shown in the coronal section adapted from Paxinos and Watson Rat Brain At-las. (**B**) AAV-shNMU infected neurons in the LH fluoresce green 17 days after viral injection. Scale bar indicates 200 µm.
