# Supplementary material for: Examining the Role of Hypothalamus-Derived Neuromedin-U (NMU) in Bone Remodeling of Rats
Source: Life (Basel). 2023 Mar 31;13(4):918. doi: 10.3390/life13040918 (PMC10144869; doi:10.3390/life13040918)
Supplement: Supplementary file 1 [file life-13-00918-s001.zip › life-2241926-supplementary.pdf]

life

Communication

Examining the Role of Hypothalamus-Derived Neuromedin-U (NMU) in Bone Remodeling of Rats

Gabriella Born-Evers [1,2,†], Ashley L. Orr [1,2,†], Elizabeth Q. Hulsey [1,2], Maria E. Squire [3], Julia M. Hum [1,2], Lilian Plotkin [4,5], Catherine Sampson [6], Jonathan Hommel [6] and Jonathan W. Lowery [1,2,5,7,*]

1 Division of Biomedical Science, College of Osteopathic Medicine, Marian University, 3200 Cold Spring Rd, Indianapolis, IN 46222, USA; gborn502@marian.edu (G.B.-E.); adaniel348@marian.edu (A.L.O.); ehulsey329@marian.edu (E.Q.H.); jmhum@marian.edu (J.M.H.)
2 Bone & Muscle Research Group, Marian University, Indianapolis, IN 46222, USA
3 Department of Biology, The University of Scranton, Scranton, PA 18503, USA; maria.squire@scranton.edu
4 Department of Anatomy, Cell Biology & Physiology, Indiana University School of Medicine, Indianapolis, IN 46222, USA; lplotkin@iupui.edu
5 Indiana Center for Musculoskeletal Health, Indiana University School of Medicine, Indianapolis, IN 46222, USA
6 Department of Pharmacology and Toxicology, University of Texas Medical Branch, Galveston, TX 77555, USA; casampso@utmb.edu (C.S.); jdhommel@utmb.edu (J.H.)
7 Division of Academic Affairs, Marian University, Indianapolis, IN 46222, USA
* Correspondence: jlowery@marian.edu; Tel./Fax: +01-317-955-6621
† These authors contributed equally to this work.

Citation: Born-Evers, G.; Orr, A.L.; Hulsey, E.Q.; Squire, M.E.; Hum, J.M.; Plotkin, L.; Sampson, C.; Hommel, J.; Lowery, J.W. Examining the Role of Hypothalamus-Derived Neuromedin-U (NMU) in Bone Remodeling of Rats. *Life* **2023**, *13*, x. https://doi.org/10.3390/xxxxx

Academic Editor: Peter Zioupos

Received: 13 February 2023
Revised: 17 March 2023
Accepted: 25 March 2023
Published: 31 March 2023

Copyright: © 2023 by the authors. Submitted for possible open access publication under the terms and conditions of the Creative Commons Attribution (CC BY) license (https://creativecommons.org/licenses/by/4.0/).

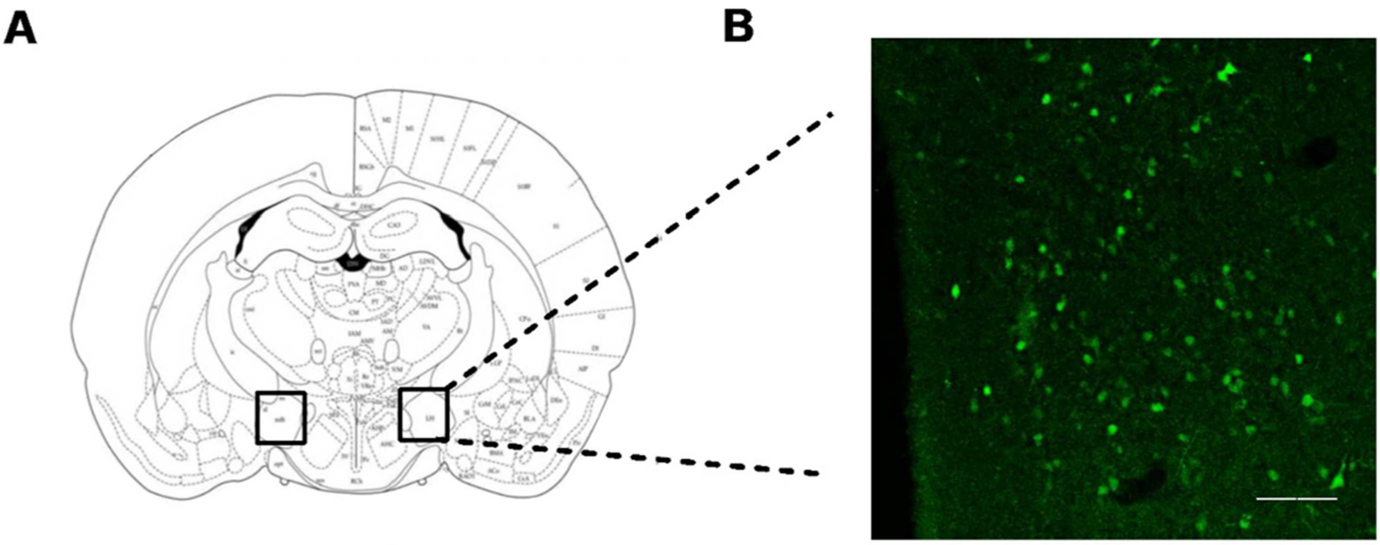

Figure S1. Targeting and knockdown efficiency of *Nmu* in the LH. (**A**) The lateral hypothalamus has previously been identified as a source of *Nmu* for the PVN and is shown in the coronal section adapted from Paxinos and Watson Rat Brain Atlas. (**B**) AAV-shNMU infected neurons in the LH fluoresce green 17 days after viral injection. Scale bar indicates 200 μm.
